# Fondaparinux versus argatroban for the management of suspected or confirmed heparin-induced thrombocytopenia: a propensity score–matched cohort study

**DOI:** 10.3389/fmed.2026.1794887

**Published:** 2026-05-08

**Authors:** Lama Alfehaid, Khalid Al Sulaiman, Abdulmajeed M. Alshehri, Saleh Alyousef, Sarah Alotaibi, Weam Abualghaith, Raghad Alsabr, Ramesh Vishwakarma, Nada Alsuhebany, Abdulrahman I. Alshaya, Shmeylan Al Harbi

**Affiliations:** 1 Department of Pharmacy Practice, College of Pharmacy, King Saud Bin Abdulaziz University for Health Sciences, Riyadh, Saudi Arabia; 2 Department of Pharmaceutical Care, King Abdulaziz Medical City, Riyadh, Saudi Arabia; 3 King Abdullah International Medical Research Center, Riyadh, Saudi Arabia; 4 Saudi Critical Care Pharmacy Research (SCAPE) Platform, Riyadh, Saudi Arabia; 5 Saudi Society for Multidisciplinary Research Development and Education (SCAPE Society), Riyadh, Saudi Arabia; 6 Norwich Medical School, University of East Anglia, Norwich, United Kingdom

**Keywords:** argatroban, critically ill patients, fondaparinux, heparin-induced thrombocytopenia, non-heparin anticoagulants, propensity score matching

## Abstract

**Background:**

Heparin-induced thrombocytopenia (HIT) is a prothrombotic, immune-mediated adverse drug reaction associated with significant morbidity and mortality. While argatroban is guideline-recommended, fondaparinux is increasingly used off-label; however, comparative real-world data remain limited, particularly in critically ill populations.

**Objective:**

To compare the effectiveness and safety of fondaparinux versus argatroban in patients with suspected or confirmed HIT.

**Methods:**

This retrospective cohort study included adult patients treated with fondaparinux or argatroban for suspected or confirmed HIT. Argatroban was administered as a continuous intravenous infusion with activated partial thromboplastin time (aPTT)-guided titration, whereas fondaparinux was given as fixed subcutaneous dosing. Propensity score matching (1:1) was performed using clinically relevant baseline variables. Outcomes included bleeding, thrombotic events, platelet recovery, and mortality.

**Results:**

Among 148 eligible patients, 96 received fondaparinux and 52 argatroban. After propensity score matching, 94 patients (47 per group) were included. Of these, 33 (22.3%) had confirmed HIT based on PF4 ELISA positivity, while 115 (77.7%) were classified as suspected HIT, defined as patients with negative or unavailable PF4 testing but clinical features consistent with HIT, including platelet count dynamics, with a similar distribution between groups. Overall, 28 patients (29.8%) were hemodynamically unstable at anticoagulation initiation. Baseline characteristics were generally well balanced after matching, although serum creatinine remained higher in the argatroban group. No statistically significant differences were observed between fondaparinux and argatroban in major bleeding (4.3% vs. 12.8%; adjusted OR 4.03, 95% CI 0.64–25.49), thrombotic events (2.1% vs. 10.6%; adjusted OR 5.07, 95% CI 0.55–46.48), or platelet recovery (87.2% vs. 76.6%; adjusted OR 0.36, 95% CI 0.11–1.19). ICU mortality was numerically higher in the argatroban group (40.4% vs. 19.1%; adjusted OR 3.02, 95% CI 1.14–7.96), whereas in-hospital mortality and length of stay were comparable.

**Conclusion:**

Fondaparinux demonstrated comparable safety and effectiveness to argatroban in patients with suspected or confirmed HIT. The higher ICU mortality observed in the argatroban group may reflect residual confounding related to illness severity and renal dysfunction. These findings suggest that fondaparinux may be a reasonable alternative in selected patients, while emphasizing individualized anticoagulant selection.

## Introduction

Heparin-induced thrombocytopenia (HIT) is a serious, immune-mediated adverse drug reaction that can occur with any form of heparin exposure, regardless of dose, frequency, or route of administration, including both unfractionated heparin (UFH) and low molecular weight heparin (LMWH) (1). The pathogenesis involves the formation of platelet-activating IgG antibodies against complexes of heparin and platelet factor 4 (PF4), triggering intense platelet activation and paradoxical thrombosis despite thrombocytopenia (2). If untreated, HIT can result in mortality rates approaching 20%; however, early recognition and prompt initiation of appropriate anticoagulation can reduce this to below 2% (3).

Optimal management requires the immediate cessation of all heparin products and initiation of a non-heparin anticoagulant in patients with an intermediate or high clinical probability of HIT (4Ts score > 4). The 2018 American Society of Hematology (ASH) guidelines recommend several options: argatroban, bivalirudin, danaparoid, fondaparinux, or a direct oral anticoagulant (DOAC), with the choice guided by drug availability, organ function, bleeding risk, pharmacokinetic profile, and hemodynamic status (1). Importantly, among these, only danaparoid and argatroban are formally approved for HIT treatment in many jurisdictions (4, 5).

Fondaparinux, a synthetic factor Xa inhibitor, is commonly used off-label for HIT because it can be given subcutaneously, does not require routine monitoring, and is generally well tolerated and cost-effective (5–7). Earlier data were largely limited to small retrospective studies, case reports, and one prospective cohort comparing fondaparinux with historical lepirudin-treated controls (8–11). Since then, additional observational studies, including propensity score (PS)–matched analyses and systematic reviews, have evaluated fondaparinux in HIT (12–16). Overall, these studies suggest comparable safety and effectiveness to direct thrombin inhibitors, although variations in study design and patient populations make direct comparisons across studies challenging. In addition, data from Middle Eastern populations remains limited. This emphasizes the need for further real-world evidence, particularly in higher-risk populations and in direct comparisons with commonly used agents such as argatroban (14, 15).

In clinical practice, patients with greater illness severity or hemodynamic instability are often managed with intravenous, rapidly titratable anticoagulants such as argatroban or bivalirudin, consistent with current guideline recommendations (1). However, fondaparinux is increasingly used beyond these traditional boundaries, including in critically ill or unstable patients, driven by its convenience and local availability.

At our institution, both fondaparinux and argatroban are routinely used for the management of suspected or confirmed HIT across a broad spectrum of clinical presentations. This provides a unique opportunity to evaluate their comparative effectiveness in a real-world setting that includes both hemodynamically stable and unstable patients. Accordingly, this study aims to compare the safety and effectiveness of fondaparinux versus argatroban in a contemporary cohort, offering additional insight into anticoagulant selection across varying levels of clinical severity.

## Materials and methods

### Study design and setting

This single-center retrospective study was conducted at King Abdulaziz Medical City, Ministry of National Guard Health Affairs (MNGHA), Riyadh, Saudi Arabia. Adult patients (≥18 years), including those admitted to the ICU, who received fondaparinux or argatroban for suspected HIT between January 1, 2016, and June 30, 2025, were eligible for inclusion. Patients were identified using electronic pharmacy dispensing and medication administration records, which were cross-referenced with laboratory orders for HIT enzyme-linked immunosorbent assay (ELISA) to confirm evaluation for HIT. The study was approved by the Institutional Review Board at King Abdullah International Medical Research Center (KAIMRC) (NRR24/037/9).

### Study subjects

All adult patients who received at least one dose of fondaparinux or argatroban for suspected or confirmed HIT during the study period were screened. In routine clinical practice, the diagnosis of HIT was based on a combination of clinical probability assessment and laboratory testing. Confirmed HIT was defined as a positive PF4/heparin ELISA result. Functional platelet activation assays were not available at our institution. Suspected HIT was defined as an intermediate or high 4Ts score (≥4) with clinical features consistent with HIT, including a platelet count fall ≥ 50% or 30%–50% temporally related to heparin exposure and recovery following heparin discontinuation, in the setting of negative or unavailable PF4 testing, as assessed by a consultant hematologist. In all cases, the decision to initiate non-heparin anticoagulation was made in consultation with the hematology service. In line with guideline-based practice, treatment was often initiated based on clinical probability prior to laboratory confirmation, particularly given potential delays in PF4 ELISA turnaround time within our institution.

Patients were excluded if they had active bleeding unrelated to HIT, contraindications to anticoagulation, or receipt of alternative non-heparin anticoagulants as part of HIT management.

Heparin-induced thrombocytopenia laboratory testing was performed using a PF4/heparin ELISA. A polyspecific immunoassay detecting IgG, IgM, and IgA antibodies (Asserachrom HPIA, Diagnostica Stago, Asnières-sur-Seine, France) was used, with results reported as optical density (OD) values. An OD threshold of ≥1.0 was considered positive per manufacturer recommendations. Functional confirmatory assays, such as the serotonin release assay (SRA) or heparin-induced platelet activation (HIPA) assay, were not routinely available.

### Study data

Collected data included demographic characteristics (age, sex, and smoking history), baseline laboratory values (serum creatinine, hemoglobin, hematocrit, and platelet count), and relevant comorbidities, including diabetes mellitus, chronic kidney disease, and acute kidney injury (AKI). AKI was defined according to the Kidney Disease: Improving Global Outcomes (KDIGO) criteria based on changes in serum creatinine, specifically as an increase of ≥0.3 mg/dL within 48 h or ≥1.5 times baseline within 7 days (17). Urine output criteria were not consistently available and were therefore not included in the definition. Additional comorbidities included liver disease (classified according to the Child–Pugh score), heart failure (with documented ejection fraction), autoimmune disease, prior arterial or venous thrombosis, hematologic malignancy, and the Charlson Comorbidity Index. Information on pre-admission use of oral anticoagulants (e.g., warfarin, apixaban, rivaroxaban) and antiplatelet agents (e.g., aspirin, clopidogrel) was also collected.

Heparin-related variables included the type of heparin exposure (UFH or LWMW), the dosing indication (therapeutic or prophylactic), the duration of exposure, and the timing of discontinuation. Laboratory testing results, including PF4 immunoassay findings, were recorded when available. Platelet kinetics were comprehensively documented, including baseline platelet count, timing and magnitude of platelet decline, nadir values, and recovery trends. These parameters were used to support clinical assessment and validation of the 4Ts score rather than as independent analytical variables. The 4Ts score was captured to assess the pretest probability of HIT. The interval between clinical suspicion of HIT and initiation of non-heparin anticoagulation was recorded when available and included in the clinical assessment. Due to variability in documentation, this variable was not included as a primary analytical measure.

Treatment-related data included the non-heparin anticoagulant used (fondaparinux or argatroban), dosing strategy, duration of therapy, and treatment modifications. Hemodynamic instability was defined as the presence of shock, use of vasopressors or inotropes, or requirement for mechanical circulatory support within ±24 h of anticoagulant initiation. This variable was treated as a baseline clinical characteristic and covariate in the analysis. Switching between non-heparin anticoagulants prior to platelet recovery was also documented.

Argatroban dosing was adjusted based on activated partial thromboplastin time (aPTT) measured using Actin FSL reagent (Siemens Healthineers) on a Sysmex CS-series coagulometer.

Platelet recovery was defined as a platelet count ≥ 100 × 10?/L or return to baseline platelet count. Recovery was assessed from the time of anticoagulant initiation through hospitalization and up to hospital discharge. Transition to oral anticoagulant therapy following platelet recovery (e.g., apixaban, dabigatran, or warfarin) was documented, including the timing of initiation.

Concomitant interventions, including thrombolysis, surgical procedures, and mechanical circulatory support, were collected from medical records when available. Due to the low frequency of these interventions, they were not included in the primary multivariable or propensity score (PS) models to avoid model overfitting; however, they were assessed descriptively.

### Clinical outcomes

Efficacy outcomes included the incidence and timing of thrombotic complications [e.g., deep vein thrombosis (DVT), pulmonary embolism (PE), myocardial infarction, and stroke], ischemic events (e.g., gangrene and bowel ischemia), and thrombus propagation. Thrombotic complications were confirmed using standard imaging modalities, including duplex ultrasonography for DVT, computed tomography pulmonary angiography for PE, and other appropriate diagnostic imaging based on the clinical scenario. Only new or clinically progressive thrombotic events occurring after initiation of anticoagulant therapy were included. Events were identified from clinical documentation and radiologic reports and were assessed from treatment initiation until hospital discharge. Thrombus propagation was defined as new or worsening thrombotic events at the same or new vascular sites, confirmed by appropriate imaging modalities and documented in radiologic reports.

Safety outcomes included major bleeding and clinically relevant non-major bleeding (CRNMB), as defined by the International Society on Thrombosis and Hemostasis (ISTH) (18, 19), as well as transfusion requirements. Additional outcomes included ICU length of stay, hospital length of stay, ICU mortality, and in-hospital mortality.

### Statistical analysis

Continuous variables were summarized as mean with standard deviation (SD) or median with interquartile range (Q1–Q3), as appropriate based on data distribution, and were compared using the Student’s *t*-test or Mann–Whitney U test, respectively. Categorical variables were presented as frequencies and percentages and compared using the chi-square test or Fisher’s exact test, as appropriate.

Propensity score matching was performed to reduce baseline differences between patients treated with fondaparinux and those receiving argatroban. Patients were matched in a 1:1 ratio using a greedy nearest-neighbor algorithm without replacement. PS were estimated using variables selected *a priori* based on clinical relevance to HIT management and outcomes, including age, location at HIT onset (ICU vs. non-ICU), presence of acute kidney injury, baseline 4Ts score, and use of mechanical circulatory support. Variables such as serum creatinine and renal replacement therapy were not directly included in the propensity model due to their strong collinearity with treatment selection and potential for structural confounding by indication.

After PS matching, outcomes were compared between treatment groups using standard statistical methods. Matching was used to improve baseline comparability; however, analyses were not explicitly modeled as paired comparisons.

Multivariable logistic regression was used to estimate associations between treatment groups and clinical outcomes, with results reported as odds ratios (ORs) and 95% confidence intervals (CIs). In models with sparse data or evidence of complete or quasi-complete separation, Firth penalized logistic regression was employed to reduce small-sample bias and improve estimate reliability. Standard logistic regression was applied when model assumptions were satisfied, and event counts were adequate.

Missing data were handled using a complete-case analysis approach. Given the retrospective design and the relatively low proportion of missing data, imputation was not performed. Multiple imputation was not pursued due to the modest sample size and potential violation of imputation assumptions, which could introduce additional bias. A two-sided *P*-value < 0.05 was considered statistically significant. All statistical analyses were conducted using SAS version 9.4 (SAS Institute Inc., Cary, NC, USA).

## Results

**FIGURE 1:**
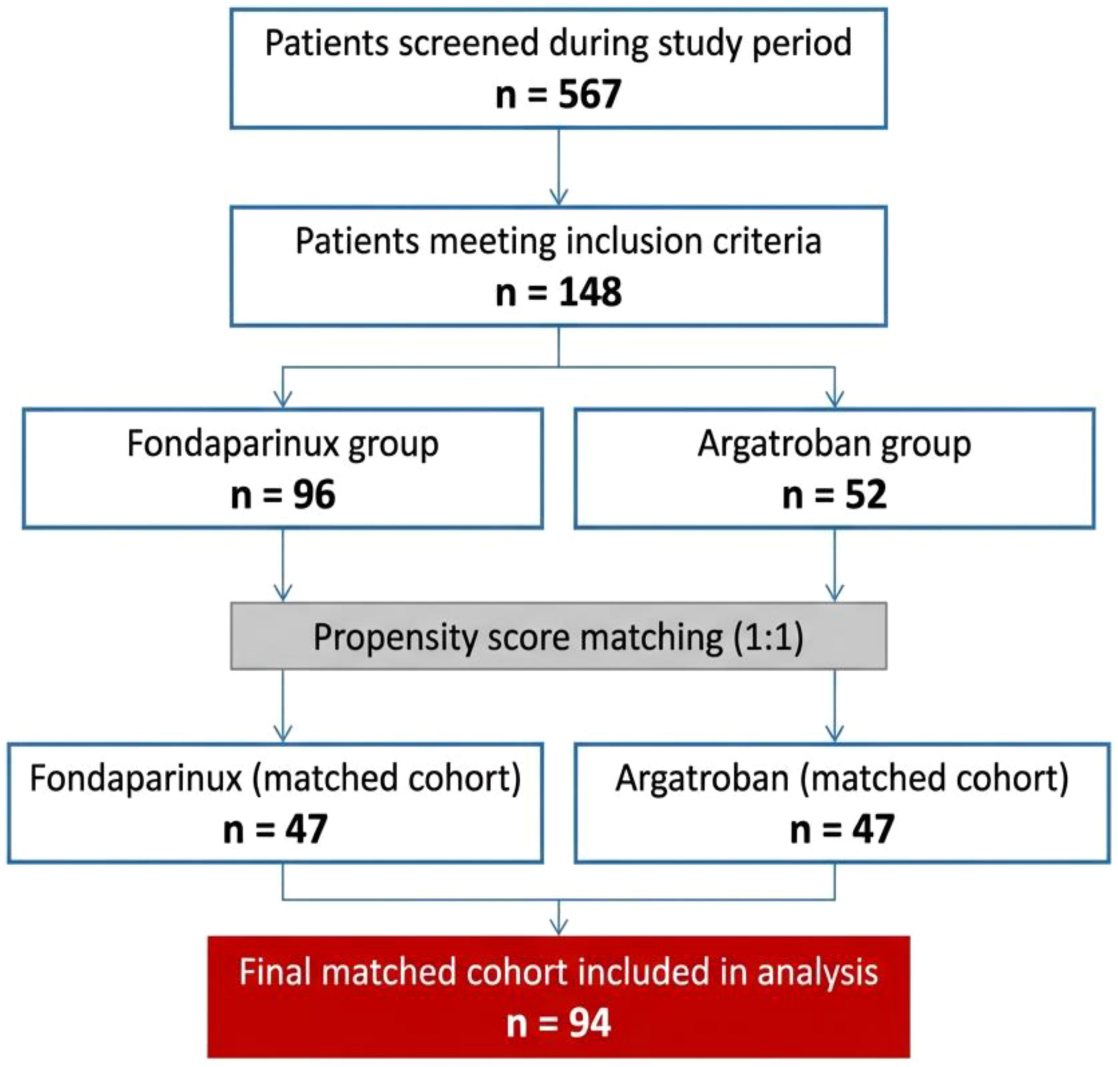
Study flowchart of included and excluded patients. Flow chart showing patient screening, inclusion, treatment allocation, and propensity score–matched cohorts. A total of 567 patients were screened, of whom 148 met the inclusion criteria. After 1:1 propensity score matching, 94 patients were included in the final analysis, with 47 per treatment group.

During the study period, of 567 screened patients, 148 met inclusion criteria. Ninety-six patients received fondaparinux and 52 received argatroban. After 1:1 PS matching, 94 patients were included in the final analysis, with 47 per treatment group (Figure 1).

Dosing strategies for both agents were consistent with contemporary guideline recommendations (1, 4). Fondaparinux dosing was individualized according to renal function and body weight. Argatroban was administered as a continuous intravenous infusion and titrated to achieve a target aPTT of 1.5–3.0 times baseline, in accordance with institutional protocols. In critically ill patients, conservative initial argatroban dosing strategies were frequently employed due to concerns regarding bleeding risk and clinical instability. Detailed data on time to therapeutic aPTT and proportion of time within the therapeutic range were not consistently available and therefore could not be reliably analyzed.

### Baseline characteristics

Baseline demographic, clinical, and laboratory characteristics of the study population before and after propensity score (PS) matching are summarized in Table 1. The median age of the overall cohort was 67 years (IQR, 58–79) and was similar between the two groups. Male patients predominated, with no significant difference between groups.

**TABLE 1:** Baseline characteristics before and after propensity score matching.

Characteristic	Before PS matching overall (*N* = 148)	Fondaparinux (*N* = 96)	Argatroban (*N* = 52)	*P*-value	After PS matching overall (*N* = 94)	Fondaparinux (*N* = 47)	Argatroban (*N* = 47)	*P*-value
Demographics								
Age, median (Q1–Q3)	64.5 (57–80)	64.0 (52–84)	65.0 (59–79)	0.81	67.0 (58–79)	67.0 (58–79)	64.5 (57–79)	0.69
Male sex, *n* (%)	85 (57.4)	58 (60.4)	27 (51.9)	0.32	52 (55.3)	29 (61.7)	23 (48.9)	0.21
Female sex, *n* (%)	63 (42.6)	38 (39.6)	25 (48.1)	0.32	42 (44.7)	18 (38.3)	24 (51.1)	0.21
BMI, median (Q1–Q3)	28.6 (24.0–33.0)	27.9 (23.5–32.1)	29.9 (25.4–34.1)	0.18	28.8 (25.4–34.0)	27.6 (25.3–33.0)	29.8 (25.4–34.1)	0.51
HIT classification, n (%)								
Confirmed HIT (PF4-positive)	33 (22.3)	15 (15.6)	18 (34.6)	0.01	24 (25.5)	10 (21.3)	14 (29.8)	0.16
Suspected HIT	115 (77.7)	81 (84.4)	34 (65.4)		70 (74.5)	37 (78.7)	33 (70.2)	
Comorbidities, n (%)								
Heart failure	34 (23.0)	18 (18.8)	16 (30.8)	0.10	21 (22.3)	7 (14.9)	14 (29.8)	0.08
Myocardial infarction	2 (1.4)	2 (2.1)	0 (0.0)	0.29	1 (1.1)	1 (2.1)	0 (0.0)	0.31
Hypertension	86 (58.1)	57 (59.4)	29 (55.8)	0.67	55 (58.5)	30 (63.8)	25 (53.2)	0.30
Diabetes mellitus	82 (55.4)	51 (53.1)	31 (59.6)	0.45	54 (57.4)	27 (57.4)	27 (57.4)	>0.99
Chronic kidney disease	32 (21.6)	15 (15.6)	17 (32.7)	0.02	26 (27.7)	12 (25.5)	14 (29.8)	0.64
Deep vein thrombosis	11 (7.4)	4 (4.2)	7 (13.0)	0.04	7 (7.4)	1 (2.1)	6 (12.8)	0.05
Pulmonary embolism	9 (6.1)	5 (5.2)	4 (7.7)	0.55	8 (8.5)	4 (8.5)	4 (8.5)	>0.99
Arterial thrombosis	23 (15.5)	15 (15.6)	8 (15.4)	0.97	14 (14.9)	6 (12.8)	8 (17.0)	0.56
Atrial fibrillation	20 (13.5)	10 (10.4)	10 (19.2)	0.13	14 (14.9)	5 (10.6)	9 (19.1)	0.25
Liver disease	11 (7.4)	7 (7.3)	4 (7.7)	0.93	7 (7.4)	4 (8.5)	3 (6.4)	0.69
Baseline clinical status at initiation								
Hemodynamic instability†	30 (20.3)	18 (18.8)	12 (23.1)	0.52	28 (29.8)	15 (31.9)	13 (27.7)	0.65
Hemodialysis or CRRT	19 (12.8)	3 (3.1)	16 (30.8)	<0.01	16 (17.0)	2 (4.3)	14 (29.8)	<0.01
Acute kidney injury	40 (27.0)	18 (18.8)	22 (42.3)	<0.01	31 (33.0)	13 (27.7)	18 (38.3)	0.27
Laboratory values, median (Q1–Q3)								
Hemoglobin	93 (86–109)	95 (88–116)	91 (82–103)	0.01	91 (82–104)	91 (83–105)	91 (82–103)	0.64
Platelets	86.5 (65–126)	92.5 (70–134)	81.5 (63–105)	0.13	82 (63–123)	82 (63–144)	82 (61–112)	0.50
Serum creatinine	86.5 (65–141)	72.5 (56–95)	129.5 (83–229)	<0.01	95 (65–156)	72 (52–95)	129.5 (83–196)	<0.01
4Ts score	4 (3–5)	3 (2–5)	4 (4–5.5)	<0.01	4 (4–6)	4 (3–6)	4 (4–5)	0.79
Charlson Comorbidity Index	4 (2–6)	4 (2–5.5)	4 (2–6)	0.62	4 (2–6)	4 (2–6)	4 (2–6)	0.91

Continuous variables are presented as median (Q1–Q3); categorical variables as *n* (%). *P*-values were calculated using Student’s *t*-test, Wilcoxon rank-sum test, chi-square test, or Fisher’s exact test, as appropriate. Confirmed HIT was defined as PF4-positive. Suspected HIT was defined based on clinical criteria with negative or unavailable PF4 testing. Percentages are calculated using the total cohort as denominator. CRRT, Continuous renal replacement therapy. †Hemodynamic instability defined as shock, vasopressor/inotrope use, or mechanical circulatory support within ±24 h of anticoagulant initiation.

Prior to PS matching, hypertension was the most common comorbidity, followed by diabetes mellitus, heart failure, and chronic kidney disease (CKD). CKD and deep vein thrombosis (DVT) were more frequent in the argatroban group compared with the fondaparinux group (32.7% vs. 15.6%, *p* = 0.016; and 13.0% vs. 4.2%, *p* = 0.04, respectively), whereas other comorbidities and the Charlson Comorbidity Index were similar between groups. Hemodynamic instability at the time of anticoagulant initiation was observed in 20.3% (*n* = 30) of patients overall, including 18.8% (*n* = 18) in the fondaparinux group and 23.1% (*n* = 12) in the argatroban group.

After PS matching, baseline demographic characteristics, comorbidities, and hemodynamic status were well balanced between groups, with no statistically significant differences observed. The proportion of hemodynamically unstable patients was 29.8% (*n* = 28) overall, including 31.9% (*n* = 15) in the fondaparinux group and 27.7% (*n* = 13) in the argatroban group. Advanced interventions, including thrombolysis, surgical procedures, and mechanical circulatory support, were infrequent in both groups.

Overall, 33 patients (22.3%) had confirmed HIT based on PF4 ELISA positivity, while 115 (77.7%) were classified as suspected HIT, defined as patients with negative or unavailable PF4 testing but clinical features consistent with HIT. The distribution of HIT classification was similar between treatment groups after matching (Table 1).

At the time of initiation of non-heparin anticoagulants, AKI was more common in the argatroban group than in the fondaparinux group (42.3% vs. 18.8%; *p* = 0.002). The median baseline 4Ts score was also higher in the argatroban group (4 vs. 3; *p* = 0.001). Baseline laboratory parameters, including serum creatinine and PF4 positivity, were higher in the argatroban group, whereas hemoglobin and hematocrit levels were higher in the fondaparinux group. After PS matching, most clinical and laboratory variables were comparable between groups; however, serum creatinine remained significantly higher in the argatroban group (Table 1).

### Clinical outcomes after propensity score matching

#### Major and CRNM bleeding events

In crude analysis, major bleeding occurred in six patients in the argatroban group and two patients in the fondaparinux group (12.8% vs. 4.3%; *p* = 0.139). Regression analysis showed a similar direction of effect, although not statistically significant (aOR: 4.03; 95% CI: 0.63–25.48; *p* = 0.14).

Minor bleeding and CRNM bleeding were numerically higher in the argatroban group in both crude analysis (8.5% vs. 4.3%; *p* = 0.39 and 2.1% vs. 0%; *p* = 0.35, respectively) and regression analysis (aOR: 1.87; 95% CI: 0.30–11.47; *p* = 0.50 and aOR: 3.14; 95% CI: 0.28–34.65; *p* = 0.35, respectively), without reaching statistical significance (Table 2).

**TABLE 2:** Clinical outcomes after propensity score matching.

Outcome	Fondaparinux (*n* = 47)	Argatroban (*n* = 47)	*P*-value	Adjusted effect estimate	*P*-value
Bleeding events, *n* (%)					
Major bleeding†	2 (4.3)	6 (12.8)	0.14	OR 4.03 (0.64–25.49)	0.14
Minor bleeding†	2 (4.3)	4 (8.5)	0.40	OR 1.87 (0.30–11.47)	0.50
Clinically relevant non-major bleeding†	0 (0.0)	1 (2.1)	0.32	OR 3.14 (0.28–34.66)	0.35
Thrombotic events, n (%)					
New arterial or venous thrombosis	1 (2.1)	5 (10.6)	0.09	OR 5.07 (0.55–46.48)	0.15
Second thrombosis	0 (0.0)	4 (20.0)	0.07	OR 3.83 (0.28–53.35)	0.32
Other clinical outcomes, *n* (%)					
ICU mortality	9 (19.1)	19 (40.4)	0.02	OR 3.02 (1.14–7.96)	0.03
In-hospital mortality	11 (23.4)	18 (38.3)	0.12	OR 2.04 (0.81–5.16)	0.13
Platelet recovery§	41 (87.2)	36 (76.6)	0.18	OR 0.36 (0.11–1.19)	0.09
PRBC transfusion	7 (14.9)	7 (14.9)	>0.99	OR 0.90 (0.25–3.28)	0.88
Platelet transfusion	2 (4.3)	3 (6.4)	0.65	OR 1.53 (0.24–9.90)	0.65
Length of stay, median (Q1–Q3)					
Hospital LOS (days)	32 (13–75)	37 (20–68)	0.66	β−0.16 (−0.50 to 0.18)	0.35
ICU LOS (days)	12 (4–26)	19 (11–37)	0.15	β−0.08 (−0.63 to 0.47)	0.77

Values are presented as *n* (%) or median (Q1–Q3), as appropriate. †Defined according to ISTH criteria. §Platelet recovery defined as platelet count ≥ 100 × 10?/L or return to baseline. OR, odds ratio from multivariable logistic regression.

#### Packed red blood cell (PRBC) and platelet transfusion

Packed red blood cell transfusion rates were similar between groups in both crude analysis (7.0% vs. 7.0%; *p* > 0.99) and adjusted analysis (aOR: 0.90; 95% CI: 0.24–3.28; *p* = 0.88). Platelet transfusion was infrequent and did not differ significantly between groups, consistent with guideline recommendations to avoid routine platelet transfusion in HIT unless clinically indicated. No statistically significant differences were observed in crude analysis (4.3% vs. 6.4%; *p* = 0.65) or adjusted analysis (aOR: 1.53; 95% CI: 0.23–9.90; *p* = 0.65).

#### Thrombotic events

New arterial or venous thrombotic events after initiation of non-heparin anticoagulation were more frequent in the argatroban group compared with the fondaparinux group (10.6% vs. 2.1%; *p* = 0.09). Regression analysis demonstrated a similar trend without statistical significance (aOR: 5.07; 95% CI: 0.55–46.47; *p* = 0.15).

Similarly, four patients (20.0%) in the argatroban group developed a second thrombotic event during hospitalization, whereas no cases were observed in the fondaparinux group (*p* = 0.0748). This finding remained non-significant in regression analysis (aOR: 3.83; 95% CI: 0.27–53.34; *p* = 0.32). Platelet recovery was comparable between groups (87.2% vs. 76.6%; *p* = 0.18), with consistent findings in regression analysis (aOR: 0.36; 95% CI: 0.10–1.18; *p* = 0.09).

#### In-hospital mortality and length of stay (LOS)

ICU mortality was higher in the argatroban group compared with the fondaparinux group in crude analysis (40.4% vs. 19.1%; *p* = 0.02), with consistent findings in regression analysis (aOR: 3.02; 95% CI: 1.14–7.95; *p* = 0.026).

In-hospital mortality was also higher in the argatroban group; however, this did not reach statistical significance in crude analysis (38.3% vs. 23.4%; *p* = 0.12) or regression analysis (aOR: 2.04; 95% CI: 0.80–5.15; *p* = 0.13).

Hospital and ICU length of stay were comparable between groups in crude analysis (32.0 [IQR 13–75] vs. 37.0 [IQR 20–68]; *p* = 0.66 and 12.0 [IQR 4–26] vs. 19.0 [IQR 11–37]; *p* = 0.15, respectively). Regression analysis yielded similar results for hospital LOS (β-coefficient: −0.16; 95% CI: −0.50 to 0.18; *p* = 0.35) and ICU LOS (β-coefficient: −0.08; 95% CI: −0.08 to 0.47; *p* = 0.77) (Table 2).

Findings were consistent across unadjusted, PS–matched, and multivariable-adjusted analyses.

## Discussion

In this PS–matched cohort study, we compared fondaparinux and argatroban for the management of suspected or confirmed HIT across a mixed population of hemodynamically stable and unstable patients. Overall, no statistically significant differences were observed between the two agents in bleeding events, thrombotic complications, platelet recovery, hospital length of stay, or in-hospital mortality, although ICU mortality was higher in the argatroban group. Transfusion requirements were similar between groups, and platelet transfusion was infrequent, consistent with guideline recommendations to avoid routine platelet transfusion in HIT unless clinically indicated.

Regarding bleeding and thrombotic outcomes, our findings align with a growing body of comparative evidence evaluating fondaparinux and argatroban in HIT. Prior observational studies and PS–based analyses have generally reported similar rates of thrombosis and bleeding between fondaparinux and direct thrombin inhibitors, despite differences in patient populations and study design (11–16). For example, Kang et al. demonstrated comparable outcomes between fondaparinux and argatroban in a propensity-matched cohort (12), while Schindewolf et al. reported no significant differences in clinical outcomes across various non-heparin anticoagulants in a large multicenter registry (14). Similarly, systematic reviews have concluded that fondaparinux appears to have a safety and effectiveness profile comparable to that of approved agents, although the overall quality of the evidence remains limited (15).

However, our study extends prior work by including a mixed population of both hemodynamically stable and unstable patients, a group that has been underrepresented in earlier studies. The higher ICU mortality observed in the argatroban group contrasts with the generally comparable mortality reported in prior analyses and is most likely explained by residual confounding related to renal dysfunction and illness severity rather than a true treatment effect. Taken together, our findings are consistent with the growing body of evidence suggesting that fondaparinux may be a reasonable alternative in selected patients, while highlighting the importance of patient selection and clinical context when interpreting comparative outcomes.

Importantly, although fondaparinux was used in some hemodynamically unstable patients in our cohort, this should be interpreted as reflecting real-world practice rather than an endorsement of its routine use in this population. Current guidelines continue to favor intravenous agents in unstable patients, and treatment decisions should remain individualized.

Although fondaparinux remains an off-label option for HIT, accumulating real-world evidence supports its use, particularly in clinically stable patients, and aligns with the ASH guidelines’ conditional recommendation for selected HIT populations (1). The absence of statistically significant differences in bleeding or thrombosis in our study further supports fondaparinux as a reasonable alternative non-heparin anticoagulant in routine clinical practice.

The higher ICU mortality observed in the argatroban group represents the most notable finding of this study but should not be interpreted as evidence of a causal treatment effect. Despite PS matching, patients receiving argatroban had significantly higher serum creatinine levels and were more likely to require renal replacement therapy, reflecting advanced renal dysfunction. Renal failure and dialysis dependence are well-established predictors of ICU mortality and likely represent confounding by indication rather than drug-related harm. This prescribing pattern is clinically appropriate, as argatroban is hepatically metabolized and preferentially selected in patients with severe renal impairment or receiving renal replacement therapy, whereas fondaparinux is contraindicated in this setting (1, 4).

Although Charlson Comorbidity Index scores were similar between groups, this metric does not capture acute organ failure, the need for renal replacement therapy, or the dynamic severity of critical illness. Additionally, the absence of validated ICU severity scores (e.g., SOFA or APACHE II) limited our ability to fully adjust for baseline illness severity. Taken together, these factors strongly suggest that the observed ICU mortality difference reflects residual confounding related to renal dysfunction and critical illness burden rather than a direct effect of anticoagulant selection.

Differences in anticoagulation intensity may have also contributed to outcome trends. Argatroban dosing in critically ill patients is frequently initiated conservatively due to bleeding concerns, particularly in the presence of multiorgan failure or hepatic dysfunction. Prior studies have demonstrated that reduced initial dosing may delay achievement of therapeutic aPTT, potentially increasing early thrombotic risk during the most vulnerable phase of HIT (20). In our cohort, detailed data on time to therapeutic aPTT and proportion of time within target range were not consistently available, limiting our ability to assess whether anticoagulation exposure differed meaningfully between groups. As such, the numerically higher thrombotic rates observed in the argatroban group may reflect differences in anticoagulation intensity rather than intrinsic inferiority of argatroban.

Importantly, argatroban remains the preferred anticoagulant in several clinical scenarios, including patients with severe renal failure, those receiving renal replacement therapy, and situations requiring rapid titration or short drug half-life (1, 4). Conversely, fondaparinux offers practical advantages in selected hospitalized patients, including once-daily subcutaneous administration, predictable pharmacokinetics, minimal need for routine laboratory monitoring, and limited interference with coagulation assays, which may simplify anticoagulation management and facilitate transition to oral therapy (21–25). These properties may be advantageous in stable or improving ICU patients, but should not be interpreted as universally superior across all clinical contexts. During the study period, this cohort provides a real-world, descriptive snapshot of current management for suspected and confirmed heparin-induced thrombocytopenia (HIT) in a high-acuity setting.

This study has limitations inherent to its retrospective design, including potential selection bias and residual confounding. Although PS matching improved balance across many baseline characteristics, clinically important differences, particularly in renal function and illness severity, persisted, reflecting real-world treatment selection and confounding by indication. The preferential use of argatroban in patients with renal impairment or requiring renal replacement therapy is consistent with clinical practice but may have influenced comparative outcomes.

The relatively modest sample size limited statistical precision and the ability to detect smaller between-group differences. In addition, the extended study period (2016–2025) may have introduced temporal variability in clinical practice, including changes in ICU management, diagnostic approaches, and anticoagulant selection, which were not explicitly modeled.

Diagnostic heterogeneity is another important consideration, as both suspected and confirmed HIT cases were included. Laboratory confirmation relied primarily on PF4/polyanion ELISA without routine functional testing, which may increase the risk of misclassification. In addition, the 4Ts scoring system has recognized limitations in critically ill populations, where thrombocytopenia is often multifactorial and may reduce diagnostic specificity. Although HIT is classically mediated by antibodies against PF4–heparin complexes, alternative antigenic targets and more complex immune mechanisms have been described, reflecting the biological heterogeneity of HIT (26).

The outcomes presented, bleeding, transfusion, thrombotic events, platelet recovery, mortality, and length of stay, are intentionally descriptive and intended to characterize current clinical patterns, risks, and signals in routine practice rather than to establish causal efficacy. Where detailed time-dependent monitoring data (e.g., time to therapeutic aPTT, percent time in range) were unavailable, speculative analyses were avoided and findings were reported conservatively. Furthermore, although concomitant interventions were recorded, their infrequent occurrence precluded their inclusion in adjusted analyses, and residual confounding from unmeasured markers of disease severity cannot be excluded. Finally, subgroup analyses by hemodynamic status were limited by sample size and should be interpreted cautiously.

Despite these limitations, this study provides robust real-world comparative data on fondaparinux and argatroban across a clinically diverse population, including critically ill and hemodynamically unstable patients who are often underrepresented in prior studies. The use of PS matching, consistent findings across multiple analytic approaches, and inclusion of contemporary clinical practice enhance the validity and generalizability of our results.

Our findings contribute to the growing body of evidence supporting fondaparinux as a practical and effective alternative in selected patients with HIT, while highlighting the importance of individualized anticoagulant selection based on renal function, illness severity, and clinical context. These data offer clinically relevant insight into real-world decision-making and may help inform practice in settings where treatment options are influenced by patient complexity and resource availability.

## Conclusion

In this real-world cohort of patients treated for suspected or confirmed HIT, fondaparinux and argatroban demonstrated broadly comparable safety and effectiveness outcomes. While argatroban was more frequently used in patients with greater illness severity and renal impairment, no significant differences in major clinical outcomes were observed after adjustment.

These findings support the use of fondaparinux as a practical alternative in appropriately selected patients, particularly those who are clinically stable. At the same time, treatment decisions should remain individualized, taking into account patient-specific factors such as hemodynamic status, renal function, and overall clinical context.

Given the observational design and inherent limitations, including residual confounding and diagnostic heterogeneity, these results should be interpreted with caution. Prospective studies incorporating standardized diagnostic approaches and functional confirmatory testing are needed to further clarify the comparative effectiveness of non-heparin anticoagulants in HIT.

## Data Availability

The raw data supporting the conclusions of this article will be made available by the authors, without undue reservation.
